# Supplementary material for: Collaborative intelligence in AI: Evaluating the performance of a council of AIs on the USMLE
Source: PLOS Digit Health. 2025 Oct 9;4(10):e0000787. doi: 10.1371/journal.pdig.0000787 (PMC12510544; doi:10.1371/journal.pdig.0000787)
Supplement: S2 File — (ZIP) [file pdig.0000787.s002.zip › S2 Data Raw data files containing unprocessed question inputs and LLM outputs including transcripts of deliberations/STEP3_Question-59-1_Type-MC-NJ.docx]

**Facilitator** **[Round 1]:** A 38-year-old man comes to the office because of a 2-month history of intermittent episodes of hot flushes, chest discomfort, and excessive sweating, with associated panic. The episodes have been increasing in frequency and now are occurring daily. The patient says there is no specific precipitating cause of the episodes, but he has noticed that the episodes occur most frequently in the afternoon. He does not have any new stressors in his life. Medical history is significant for hypertension and type 2 diabetes mellitus. Medications are lisinopril and metformin. Family history is significant for hyperparathyroidism in an aunt and the death of an uncle from medullary thyroid cancer. The patient's BMI is 33 kg/m². Vital signs are temperature 36.9°C (98.4°F), pulse 74/min, respirations 18/min, and blood pressure 138/92 mm Hg. Physical examination discloses no abnormalities. Results of serum laboratory studies are shown: Calcium 9.8 mg/dL Urea nitrogen 15 mg/dL Creatinine 1.0 mg/dL Na+ 140 mEq/L K+ 3.8 mEq/L Cl− 102 mEq/L HCO3 −28 mEq/L ECG shows no abnormalities. Which of the following is the most appropriate diagnostic study at this time? (A) Plasma renin activity (B) Serum aldosterone concentration (C) Serum free metanephrine concentration (D) Serum parathyroid hormone concentration (E) No study is indicated

**AI_B:** RESTATING THE QUESTION: This is a case of a 38-year-old man who presents with symptoms such as hot flashes, chest discomfort, excessive sweating, and panic attacks. These episodes are occurring daily without any identified trigger, although the patient does note a higher frequency of these episodes in the afternoon. His past medical history includes hypertension and type 2 diabetes, and his family history is significant for hyperparathyroidism and medullary thyroid cancer. Lab studies are all within normal limits, and no abnormalities are noted during the physical exam. PLANNING: There are several assumptions that we can make regarding his symptoms. Given his medical history and symptoms, they may be related to his hypertension or diabetes, be a result of new stressors even though the patient has not reported any, or could potentially suggest a neuroendocrine disorder such as pheochromocytoma. Given the family history of endocrine disorders, the latter could be of higher priority. CLINICAL REASONING: The symptoms of hot flashes, chest discomfort, excessive sweating, and panic attacks do hint towards an endocrine disorder. Pheochromocytoma, a catecholamine-secreting tumor usually located in the adrenal medulla, often presents with hypertension and symptoms that are episodic and may not have an identifiable trigger. These symptoms can include headaches, palpitations, sweating, or anxiety, which seem consistent with this patient's presentation. CONCLUDING: Given his symptoms and the ruling out of other potential causes such as stress or complications related to hypertension or diabetes, it may be prudent to consider testing for pheochromocytoma. Family history strengthens this suspicion given the prevalence of endocrine disorders, even though the exact disorders in the family (hyperparathyroidism and medullary thyroid cancer) are not directly linked with pheochromocytoma. RESPONSE: In this case, considering his symptoms and family history of endocrine disorders, the most appropriate diagnostic study at this time would be (C) Serum free metanephrine concentration, which is the best diagnostic test to rule in or out the diagnosis of pheochromocytoma.

**AI_E:** RESTATING THE QUESTION: The patient is a 38-year-old man who is complaining of intermittent episodes of hot flushes, chest discomfort, excessive sweating, and panic for the past two months. These episodes have been frequent and occur mostly in the afternoon. He has a medical history of hypertension and type 2 diabetes mellitus and is currently taking lisinopril and metformin. His familial history is significant for hyperparathyroidism and medullary thyroid cancer. His vital signs and laboratory tests, including calcium, appear normal. Due to his symptoms, we need to decide what would be the most appropriate diagnostic study. PLANNING: Key assumptions would be that these symptoms are related to an underlying disorder. One assumption could be that the patient has a pheochromocytoma, a rare tumor of the adrenal glands that produces excessive catecholamines inducing the presented symptoms. Another assumption could be that he is experiencing panic attacks due mental stress or anxiety, but the patient stated that he is not under any new stress. Another assumption related to the family history of hyperparathyroidism would be that he could have a parathyroid problem, but his calcium levels are normal. CLINICAL REASONING: Given the description of the symptoms and his family history, our assumption is leaning more towards the presence of pheochromocytoma. This assumption is bolstered by his symptoms of hot flashes, chest discomfort, and excessive sweating, which are all indicative of excess catecholamine release, a characteristic of a pheochromocytoma. What further leads us to this assumption is the fact the episodes usually happen in the afternoon, possible due to some form of circadian variation in catecholamine release. While pheochromocytomas can sometimes cause hypertensive crises, the current management of the patient's hypertension seems to be controlling his blood pressure levels. CONCLUDING: Considering the patient's symptoms and common features of pheochromocytoma combined with a suggestive circadian pattern of symptoms, a diagnostic study evaluating the presence of this rare tumor seems to be crucial. RESPONSE: Therefore, considering the provided options, the most appropriate diagnostic study at this time would be a serum free metanephrine concentration test (Option C) to detect possible catecholamine-secreting tumors like pheochromocytomas in this patient.

**AI_D:** RESTATING THE QUESTION: The query is regarding a 38-year-old male patient with a background of hypertension and type 2 diabetes, associated with a familial history of hyperparathyroidism and medullary thyroid cancer. His current symptoms include hot flushes, chest discomfort, excessive sweating, and panic, occurring daily and typically in the afternoon. His basic metabolic panel results are within normal limits and physical examination is unremarkable. The question seeks to determine which diagnostic study would be most appropriate in this scenario, listing options of assays for plasma renin, serum aldosterone, serum free metanephrine, serum parathyroid hormone, or none at all. PLANNING: The symptoms experienced by the patient are generally associated with the release of hormones triggering the body's "fight or flight" response, so it seems beneficial to explore endocrinologic possibilities. Given the listed family history of glandular-oriented health issues, there could be a related genetic predisposition involved. Based on the aforementioned details, his symptoms could be due to pheochromocytoma, hyperparathyroidism, adrenal gland disorders, or a different endocrine condition. CLINICAL REASONING: Despite the family's history of hyperparathyroidism, the patient's normal serum calcium level makes this less likely. The evaluation of serum aldosterone and plasma renin could be associated with adrenal gland dysfunction; however, the patient's normal potassium levels and blood pressure under treatment don't point to primary hyperaldosteronism. On the other hand, his symptoms are consistent with pheochromocytoma, a rare condition involving a tumor in the adrenal glands, which results in excess levels of adrenaline/noradrenaline causing episodes of hypertension, heart palpitations, sweating, and hot flushes. Despite the family history of thyroid cancer, medullary thyroid cancer is not directly associated with pheochromocytoma; its association is through Multiple Endocrine Neoplasia (MEN) syndromes. CONCLUDING: Therefore, the patient's symptoms of intermittent hot flushes, chest discomfort, excessive sweating, and panic - daily and-mostly-in-the-afternoon episodes - strongly suggest the possibility of a pheochromocytoma. RESPONSE: The most appropriate diagnostic study at this time would be (C) serum free metanephrine concentration, given these factors. The serum free metanephrine assay is the preferred initial test for a biochemical diagnosis of pheochromocytoma.

**AI_C:** RESTATING THE QUESTION: The case presents a 38-year-old man with a history of hypertension and type 2 diabetes, now experiencing episodic hot flushes, chest discomfort, excessive sweating, and panic. These symptoms have no identifiable trigger and are happening every day, with an observed pattern of afternoon occurrence. Laboratory results and a physical examination did not reveal abnormal parameters. Family history indicates the presence of hyperparathyroidism and medullary thyroid cancer. The query is on what would be the most suitable diagnostic measure for his case, with options ranging from the assessment of various hormonal levels and related biochemical markers to the option of no further tests required. PLANNING: It is necessary to connect the clinical presentation with possible causes, to find a presumed assumption. The symptoms incline towards a hormonal etiology potentially related to catecholamine excess, commonly seen in pheochromocytoma. This is a neuroendocrine tumor that secretes excessive amounts of catecholamines, often presenting with paroxysmal symptoms. However, it is also important to consider that individuals with high body mass index (BMI), such as this patient, might have similar presentations due to metabolic syndrome. His family history suggests a possible genetic syndrome such as Multiple Endocrine Neoplasia (MEN) type 2 that includes medullary thyroid cancer and can also manifest with pheochromocytoma. As per the options given, the best diagnostic study should help verify or rule out this assumption. CLINICAL REASONING: Pheochromocytoma, considering the patient's presentation and family history, appears to be a plausible underlying cause, which is associated with MEN type 2. Pheochromocytoma often results in episodic symptoms due to intermittent secretion of catecholamines. These symptoms, which include episodes of hot flushes, chest discomfort, excessive sweating, and panic attacks, match our patient's presentation. Pheochromocytomas are diagnosed by measuring catecholamine byproducts (metanephrines) in the blood or urine. CONCLUDING: Supposing the underlying cause is pheochromocytoma, a hormonal (catecholamine) imbalance condition, the logical diagnostic procedure would be to assess indicators for such a condition. In this case, the Serum free metanephrine concentration test is the most suitable diagnostic study, as metanephrines are byproducts of catecholamine metabolism and their presence in excess in blood could indicate a pheochromocytoma. RESPONSE: The most appropriate diagnostic study at this time is (C) Serum free metanephrine concentration.

**AI_A:** The question is about a 38-year-old man who has been experiencing intermittent episodes of symptoms such as hot flushes, chest discomfort, excessive sweating, and associated panic. These episodes have been increasing in frequency and are currently happening daily, often in the afternoons, without any identifiable trigger. He also has a medical history of hypertension and type 2 diabetes, and is on treatment with lisinopril and metformin. His BMI is 33 kg/m², and he has some significant family medical history. Multiple lab values have been provided, all within normal range, and his ECG is also normal. In all this, the question is asking about what would be the most appropriate diagnostic study to conduct at this time. Clearly, the assumption here is that the symptoms being experienced by the patient are related to a potential medical condition that can be identified and diagnosed using tests and studies. An alternate possible assumption could be that the symptoms might be due to a side effect or an interaction of his current medications, a psychological condition such as an anxiety disorder, or an endocrine disorder, which may not show any abnormalities on standard tests. However, given the presenting symptoms and medical background of the patient, it would seem more likely that the symptoms are due to a potential endocrine disorder. Autoimmune disorders that induce these symptoms often fall within the endocrine spectrum, and the patient's family history of hyperparathyroidism and medullary thyroid cancer further supports this assumption. Considering his symptoms of hot flushes, excessive sweating, panic, increased heart rate which may not be well appreciated due to his antihypertensive medication (lisinopril), the likely differential would be a catecholamine-secreting tumor, namely pheochromocytoma. Pheochromocytomas are neuroendocrine tumors that originate from chromaffin cells in the adrenal medulla, and they secrete excessive amounts of epinephrine and norepinephrine. The secretion of these hormones leads to intermittent episodes of hypertension, palpitations, headache, excessive sweating - accounting for his symptoms. Such a condition can't be identified through conventional biochemistry lab results. Even though he has a family history of hyperparathyroidism and medullary thyroid cancer, his calcium levels are normal, which rules out hyperparathyroidism and calcium indicates that hypercalcemia secondary to a dispute in calcium metabolism is not the issue here. Therefore, a serum parathyroid hormone concentration study may not be the most helpful immediate next step. Instead, it would seem more appropriate to conduct a serum free metanephrine concentration study. Metanephrines are metabolites of catecholamines, as such, they can be used to identify the presence of a catecholamine-secreting tumor such as a pheochromocytoma. If elevated, this would confirm the suspected diagnosis. In conclusion, the most appropriate diagnostic study at this time would be a serum free metanephrine concentration study (choice C). This would help to identify a potentially catecholamine-secreting tumor such as a pheochromocytoma which may explain the patient's symptoms.

**Facilitator** The consensus among the AI responses is that the most appropriate diagnostic study for the 38-year-old man presenting with intermittent episodes of hot flushes, chest discomfort, excessive sweating, and associated panic, would be a serum free metanephrine concentration test (Option C). This conclusion is based on the patient's symptoms, medical history, and family history. The symptoms experienced by the patient, including hot flushes, chest discomfort, excessive sweating, and panic, are indicative of a potential endocrine disorder. Given the patient's family history of hyperparathyroidism and medullary thyroid cancer, the possibility of an endocrine disorder is further supported. The most likely differential diagnosis, considering the patient's symptoms and medical background, is a catecholamine-secreting tumor, namely pheochromocytoma. Pheochromocytomas are neuroendocrine tumors that originate from chromaffin cells in the adrenal medulla, and they secrete excessive amounts of epinephrine and norepinephrine. The secretion of these hormones leads to intermittent episodes of hypertension, palpitations, headache, excessive sweating - accounting for his symptoms. A serum free metanephrine concentration test is the preferred initial test for a biochemical diagnosis of pheochromocytoma. Metanephrines are metabolites of catecholamines, and their presence in excess in the blood could indicate a pheochromocytoma. Therefore, given the patient's symptoms and the ruling out of other potential causes such as stress or complications related to hypertension or diabetes, it may be prudent to consider testing for pheochromocytoma. In conclusion, the consensus is that the most appropriate diagnostic study at this time would be a serum free metanephrine concentration study. This would help to identify a potentially catecholamine-secreting tumor such as a pheochromocytoma which may explain the patient's symptoms.
